# VisPan: real-time visualisation of multiplex amplicon-based sequencing panels for rapid syndromic surveillance and pathogen detection

**DOI:** 10.1093/bioinformatics/btag351

**Published:** 2026-05-29

**Authors:** Pierre Lechat, Aurelia Kwasiborski, Rémi Vincent, Jessica Vanhomwegen, Jean-Claude Manuguerra, Valérie Caro, Véronique Hourdel

**Affiliations:** Institut Pasteur, Bioinformatics and Biostatistics Hub, 75015, Paris, France; Institut Pasteur, Université de Paris, Environment and Infectious Risks Unit (ERI), Laboratory for Urgent Response to Biological Threats (CIBU), 75015, Paris, France; Institut Pasteur, Université de Paris, Environment and Infectious Risks Unit (ERI), Laboratory for Urgent Response to Biological Threats (CIBU), 75015, Paris, France; Institut Pasteur, Université de Paris, Environment and Infectious Risks Unit (ERI), Laboratory for Urgent Response to Biological Threats (CIBU), 75015, Paris, France; Institut Pasteur, Université de Paris, Environment and Infectious Risks Unit (ERI), Laboratory for Urgent Response to Biological Threats (CIBU), 75015, Paris, France; Institut Pasteur, Université de Paris, Environment and Infectious Risks Unit (ERI), Laboratory for Urgent Response to Biological Threats (CIBU), 75015, Paris, France; Institut Pasteur, Université de Paris, Environment and Infectious Risks Unit (ERI), Laboratory for Urgent Response to Biological Threats (CIBU), 75015, Paris, France

## Abstract

**Motivation:**

Infectious diseases persist as a major global public health challenge. Diverse factors, including climate change, globalization, deforestation, human-animal interactions, lifestyle choices, and various biological factors, can contribute to their emergence and reemergence. Rapid detection and characterization of (re)emerging pathogens are therefore critical for effective outbreak management and for enhancing our understanding of epidemics by monitoring the transmission, spread, evolution, and genomics of pathogens. In this context, next-generation sequencing technologies (NGS), particularly long-read platforms such as Oxford Nanopore Technologies (ONT), have opened new avenues for real-time pathogen monitoring. However, the bioinformatics bottleneck remains a challenge, emphasizing the need for efficient, accessible, and user-friendly analysis tools.

**Results:**

Here, we present a tool adapted from the RAMPART software that enables real-time data visualisation of multiplex PCR syndromic panels combined with Oxford Nanopore sequencing. This real-time analysis enables rapid pathogen detection, from raw data acquisition to taxonomic assignment, within minutes. The interface offers dynamic visual tracking of the sequencing run and amplicon coverage, facilitating immediate insights during diagnostic workflows. Validation experiments confirmed the system’s reliability, accurately identifying all pathogens present in complex clinical or environmental samples. This tool provides an integrated, user-friendly solution for genomic pathogen surveillance in field or clinical settings.

## 1 Introduction

Emerging viral threats continue to impact human and animal health worldwide. Timely outbreak investigations and rapid detection of pathogens are critical for enabling coordinated and effective public health responses. Multiplex PCR (polymerase chain reaction) assays, combined with real-time genomic sequencing of pathogens, have become effective methods for addressing these challenges. Over the past decade, advances in Oxford Nanopore Technologies (ONT) have enhanced DNA and RNA sequencing capabilities, with major improvements in accuracy, read length, and throughput driven by the development of new versions of nanopores and motor proteins. At the same time, new base-calling algorithms have been developed and implemented. As a result, raw data can be rapidly accessed for downstream analysis in real time. In this study, we introduce VisPan, a real-time analysis and visualisation pipeline designed for multiplex syndromic panel sequencing using ONT data. VisPan enables rapid pathogen detection by providing intuitive, real-time tracking of sequencing runs and amplicon-level detection and identification. This tool bridges the gap between sequencing output and actionable clinical or epidemiological insight, significantly shortening the time from sample to result.

## 2 Approach and implementation

### 2.1 Syndromic panels and biological workflow

The laboratory has set up three syndromic panels, targeting pathogen groups associated with distinct clinical presentations: respiratory symptoms (Brinkmann 2022), haemorrhagic fever (Brinkmann 2017), vesicle-forming pathogens (Israeli 2022). Each panel comprises multiple pathogen-specific targets that can be amplified within a single multiplex assay. The number of targets per panel differs, ranging from 11 (vesicle-forming pathogens panel) to 541 (haemorrhagic fever panel) targets. Target-specific primer pairs are pooled to perform multiplex PCR. Following PCR amplification, the resulting products are sequenced using an Mk1C device (ONT). Several samples can be multiplexed after being individually amplified and barcoded.

**Figure 1:**
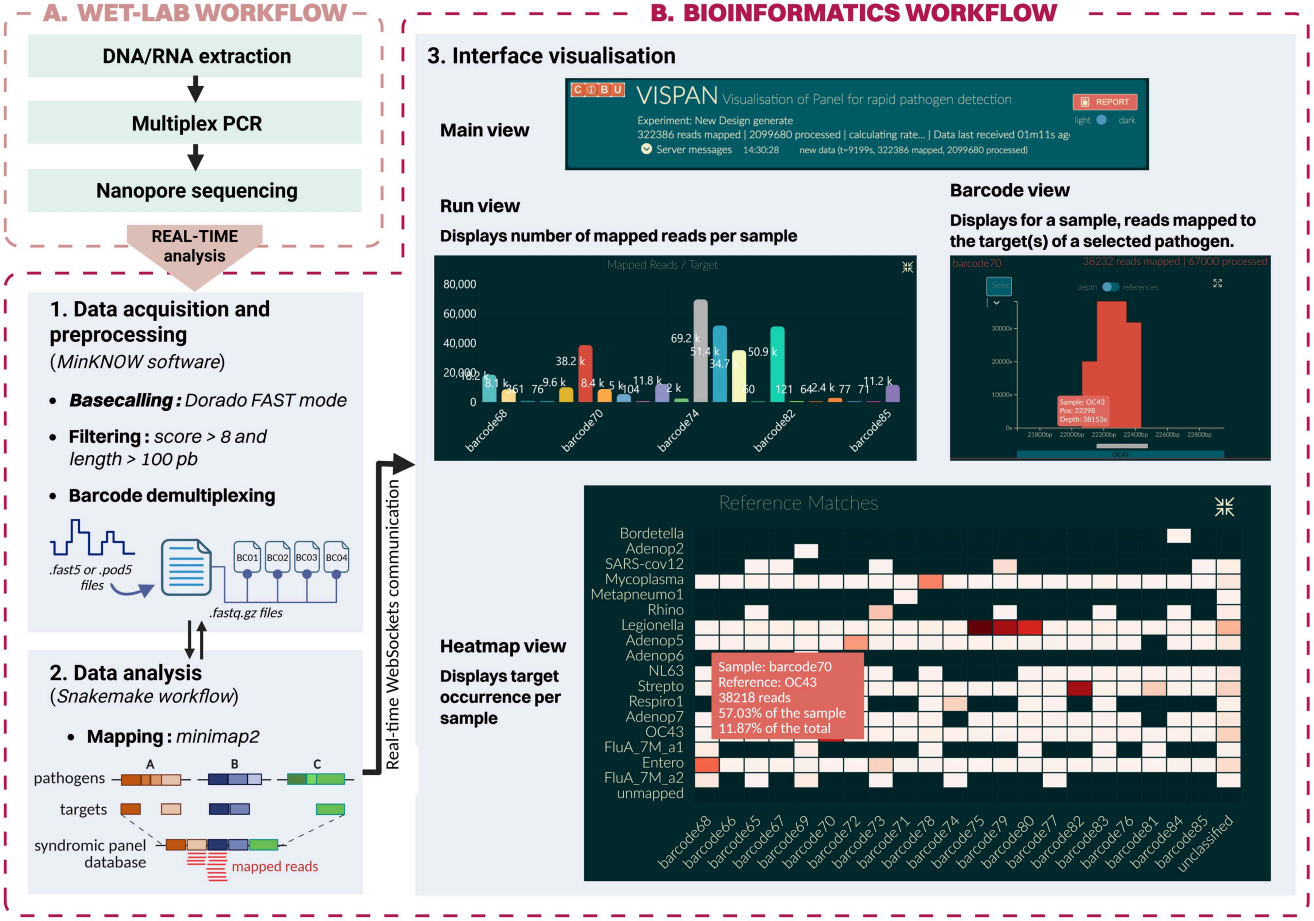
Workflows from sample preparation to real-time visualisation. (A) The wet-lab workflow begins with DNA/RNA extraction, followed by multiplex amplification and nanopore sequencing. (B) For the bioinformatics workflow, MinKNOW software manages data pre-processing (basecalling, filtering, and demultiplexing). By monitoring the sequencer’s output directory, snakemake process reads mapping using minimap2 in real-time against a dedicated syndromic panel database. Results are then transmitted via WebSockets communication, enabling immediate and dynamic visualisation as presented in the interface (run view, barcode view and heatmap view).

Figure 1A summarizes the wet-lab workflow from sample preparation to sequencing.

### 2.2 Configuration files preparation

A Python script (createPanel.py) was developed to generate the configuration files required for sequencing data visualisation. The input file (*input.txt*) is a tabular file *(.tsv* or *.txt*) containing a range of information: (i) primer sequences, (ii) target reference sequences and positions, (iii) target names. A directory (*REF_DIR*) containing the target sequences in *fasta* format (one file per target) is also required.

Output files include two configuration files in JSON format (*primers.json* and *genome.json*) and a FASTA reference database (*references.fasta*) for data analysis and visualisation. This database is used to analyse data during the mapping process. These three files must be placed in the same directory, which will be referenced when VisPan is launched. To use the script, the user must execute the following command line:


python createPanel.py−c input.txt−f REF_DIR−n name of the panel

This setup step is performed once per panel and only requires re-execution if panel content is modified.

### 2.3 Bioinformatics workflow

The bioinformatics workflow is illustrated in Fig. 1B. The ONT software MinKNOW (24.04.16) is used to control the device and converts the electrical signal (raw data in *pod5* or *fast5* format) into sequences (.*fastq* format). This step is carried out with the open source basecaller for ONT reads Dorado (7.3.11, nanoporetech 2022). Dorado offers various models based on speed and accuracy, including fast, hac (high accuracy), and sup (super-accuracy). The *fast* model is recommended because it quickly produces data in *.fastq* format (Fig. 1B.1). To view the results of real-time sequencing with VisPan, files in *.fastq.gz* format are mapped with minimap2 (2.17.r941) (Li 2018, 2021) against the appropriate database, which enables visualisation and detection of targeted pathogens present in the samples. The analysis process is automated using snakemake (Köster and Rahmann 2012), a workflow system that performs the mapping as sequences arrive in the device directory every minute (one barcode directory per sample). It then parses the barcode and mapping information and produces a csv report (Fig. 1B.2).

### 2.4 Tool implementation

VisPan tool is adapted from the stand-alone application Rampart (ARTICnetwork 2019) which provides a real-time overview of genome coverage along a linear virus, such as Ebola or SARS-CoV-2. However, visualising data from multiplex PCR sequencing panels is more difficult due to the high number of targets and pathogens. In addition, the detection of certain pathogens relies on multiple targets. For example, the respiratory panel includes three targets to detect SARS-CoV-2. In this context, new functionalities written in React have been implemented in the application. The use of this technology provides a real-time dashboard. A new data model has been implemented to assign one or more targets to a pathogen and to display this information. Our tool is organised into four views:

- The *main view* displays general information about the run (e.g. run name, date, total number of reads).
- The *run view* summarises the number of reads per sample and allows the user to select a specific sample. The barplot chart displaying the real-time reads count of the barcodes has been modified using the AMcharts API and is now more dynamic and zoomable.
- The *barcode view* features a drop-down menu allowing the search of pathogens and their associated targets. Users can zoom in to visualise the state of the mapping and to highlight specific parts of the amplified target found in the sample. This plot, encoded in D3, has been modified and adapted to the new data model. The barcode view also offers the option of exporting two files: one with read annotations and the target in .cvs format, and the other with the raw barcode data of interest in .fastq format for downstream analysis. As described in Section 2.2, reads are mapped to the relevant reference database corresponding to the syndromic panel using the minimap2 mapper.
- The *heatmap view* represents and highlights the targets present in the samples. The darker the colour, the greater the number of reads mapping to the reference. The colour range of the heatmap depends on the maximum value defined by the user in the tool parameters (reference max count).

The graphical interface is presented in Fig. 1B.3.

## 3 Evaluation and validation

To evaluate our tool, we analysed an external quality assessment (EQA) sample panel for respiratory diseases (2024 Respiratory I & II EQA from the Quality Control for Molecular Diagnostics company) using the respiratory primer panel. This EQA contains 20 blinded samples with variable pathogen composition and loads. We performed multiplex PCR in combination with sequencing on an Mk1C device. All samples and a negative control were individually barcoded and then multiplexed for sequencing (samples: barcodes 65–84; negative control: barcode 85). The results shown in the heatmap (Fig. 1B.3) illustrate our tool’s capabilities for detecting pathogens. For instance, *human beta coronavirus (OC43)* was detected in barcode 70 with 38,218 reads, representing 57.03% of the sample and 11.87% of all reads in the run. The colour difference between barcodes 75, 79, and 80, all containing *Legionella pneumophila*, correlates with the monoplex PCR cycle threshold (Ct) values of 26.32, 27.76, and 31.77, respectively. The Ct value indicates the relative level of pathogen genomic load in a sample. The higher the Ct value, the lower the genomic load and the more sensitive the detection. The number of reads mapped to *Legionella pneumophila* represents 52.76%, 47.65%, and 30.00% of the total reads for barcodes 75, 79, and 80, respectively. The complete EQA results are presented in supplementary data (Supplementary Table S1). In the negative control (barcode 85), some targets were detected with fewer than 10 reads (white colour). Overall, our tool successfully detected the pathogen present in all samples in real time. Initial results were obtained just a few minutes after the libraries were loaded on the sequencer’s flow cell (Supplementary Fig. S2). Final validation of the presence or absence of specific pathogens in each sample remains the responsibility of the experimenter. While the final interpretation of the diagnosis requires expert validation, VisPan provided real-time analytical information throughout the analysis.

## 4 Conclusion

The results of this study highlight the power of the proposed pipeline for large-scale screening of emerging pathogens and the rapid detection of infectious diseases. The Ct-correlated detection sensitivity also demonstrates VisPan’s utility for semi-quantitative assessment, a critical advantage in outbreak response. In the context of management and responses to epidemics, real-time data delivery to health authorities is both critical and essential. The developed tool is user-friendly and enables rapid and real-time analysis. Compatible with portable sequencing devices, its minimal computational requirements for both visualisation and data analysis make it well-suited for deployment in field conditions. Additionally, the configuration files are straightforward and can be easily adapted to other panels, extending VisPan’s utility across diverse outbreak scenarios.

## Supplementary Material

btag351_Supplementary_Data

## Data Availability

The program VisPan and the documentation are available at https://github.com/pasteur-cibu-detection. Python script (createPanel.py), panels configuration files (panels) can be downloaded from https://github.com/pasteur-cibu-detection. The dataset to test the application is available on Zenodo (https://zenodo.org/records/18377051). The program has also been submitted to the Software Heritage Archive at the following permalink: https://archive.softwareheritage.org/swh:1:dir:149c48e9f4291dccce8bce3ed16017733d4e8141;origin=https://github.com/PASTEUR-CIBU-detection/VisPan;visit=swh:1:snp:c7ba698e74681bc4c51017d0108cc1754a3320cc;anchor=swh:1:rev:e6bb5751f62901ffabbecec02a2272043b4e188c. License: AGPL-3. Any restrictions to use by non-academics: For commercial use and modifications, please contact the corresponding author.
