# Supplementary material for: VisPan: real-time visualisation of multiplex amplicon-based sequencing panels for rapid syndromic surveillance and pathogen detection
Source: Bioinformatics. 2026 May 29;42(6):btag351. doi: 10.1093/bioinformatics/btag351 (PMC13290484; doi:10.1093/bioinformatics/btag351)
Supplement: btag351_Supplementary_Data [file btag351_supplementary_data.docx]

**Supplementary data**

**Supp Table 1** Results of the respiratory external quality assessment (barcode/sample number, qPCR results, Ct values, total number of reads, number of mapped reads, expected pathogen, and user validation).


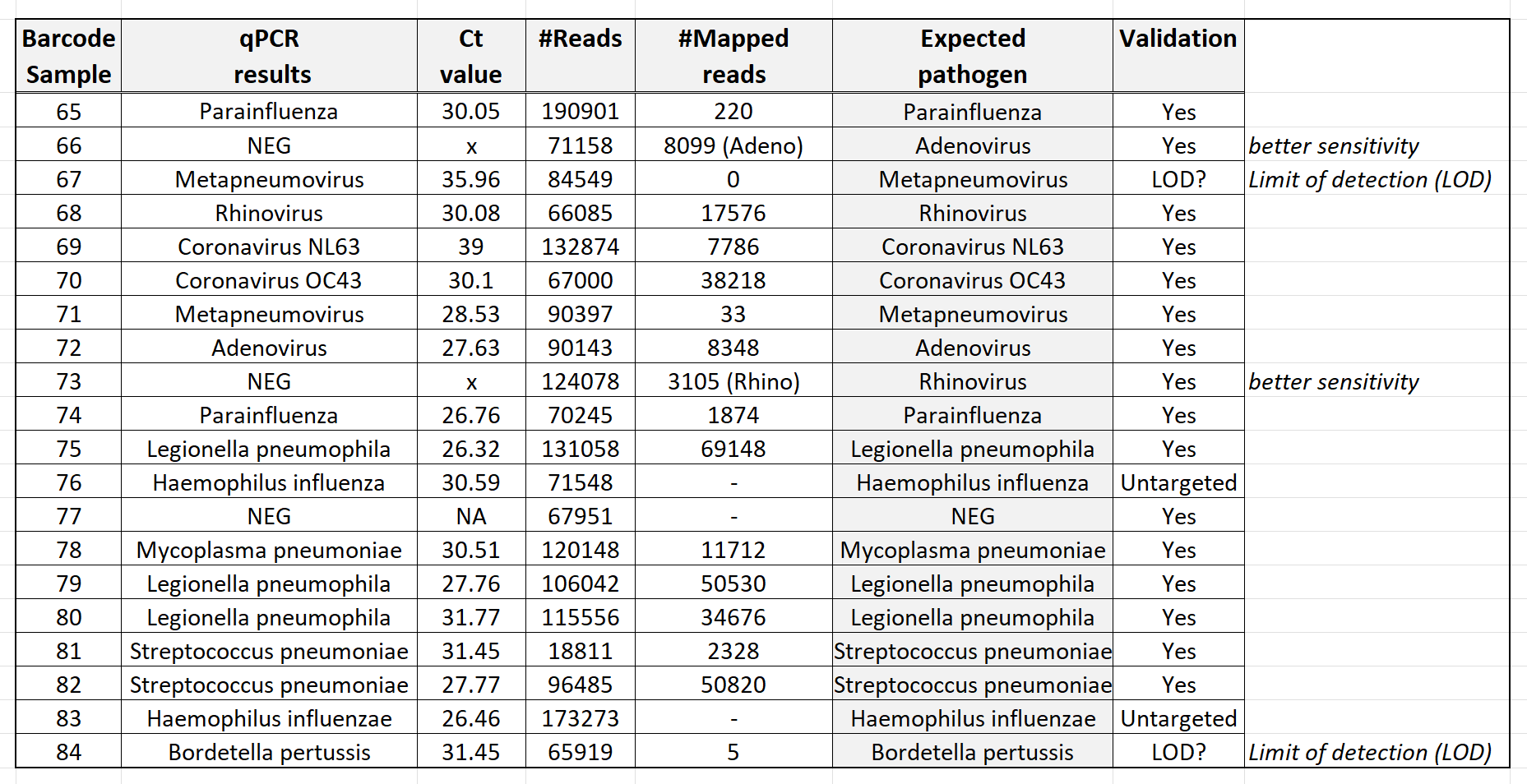


The EQA contains 20 blinded samples with variable pathogen composition and loads. All samples were individually barcoded from barcodes 65 to 84.

**Supp Figure 2**. Real-time detection of pathogen-targeted reads.


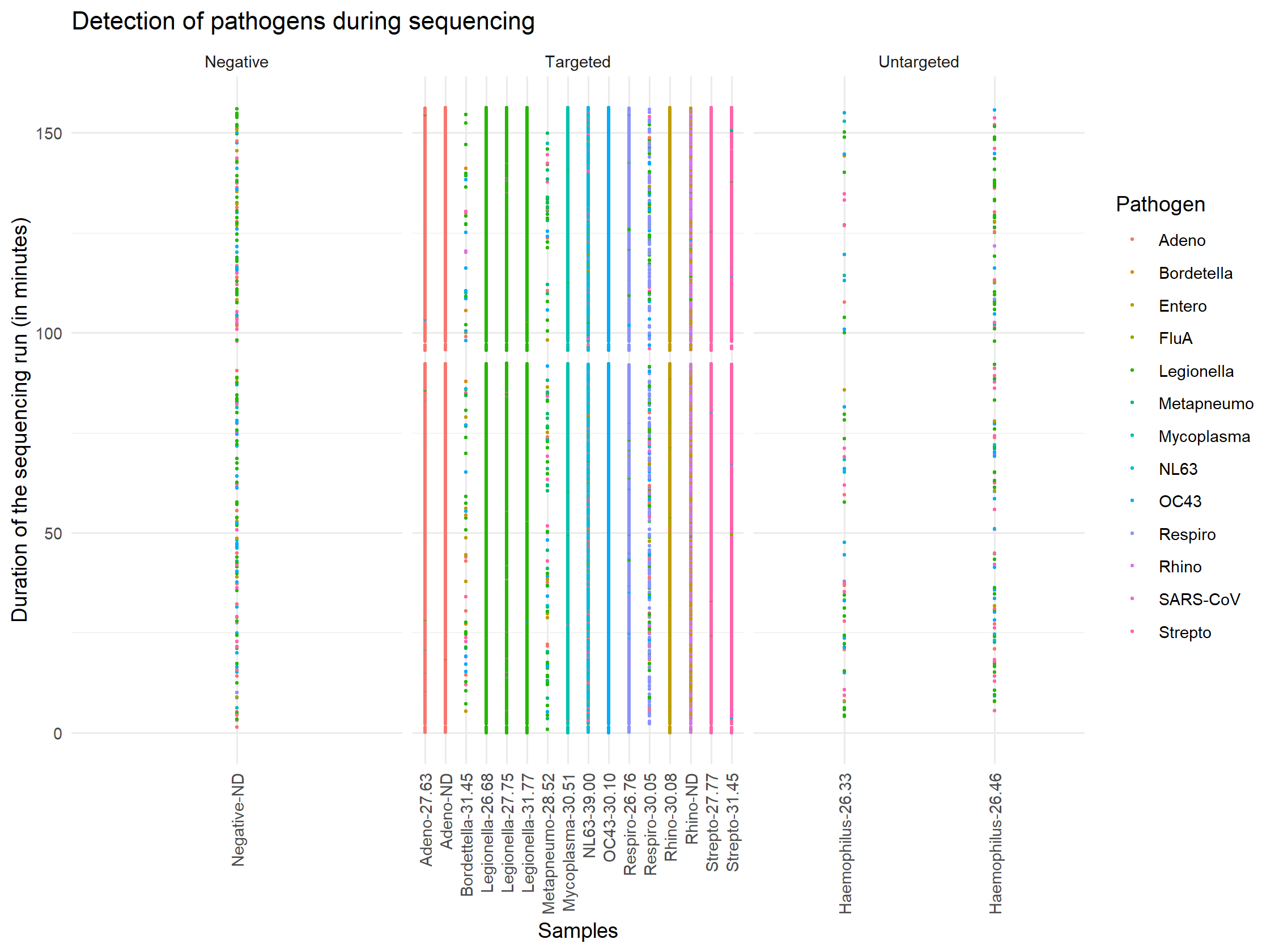


The x-axis corresponds to different samples of the EQA; the y-axis corresponds to the duration of the sequencing run (in minutes). Samples are labelled according to the pathogen detected and the corresponding Ct value of the qPCR. Samples are classified into three categories (Negative: negative control; Targeted: samples containing one pathogen targeted by the respiratory panel; and Untargeted: samples containing a pathogen not targeted by the respiratory panel. Each point of the graph corresponds to a read mapping onto a specific target for a given sequencing duration. The gap in read mapping observed at 90 min corresponds to the pore scan of the flow cell during the run. Of note, reads targeting pathogens included in the respiratory panel were detected in negative and untargeted samples, likely due to a relaxed reading of the barcodes or sequencing errors.
